# Treatment of Medication-Related Osteonecrosis of the Jaw Without and With the Use of Advanced Platelet-Rich Fibrin: A Retrospective Clinical Study

**DOI:** 10.3390/jfb16050180

**Published:** 2025-05-14

**Authors:** Paulina Adamska, Marcin Stasiak, Natalia Kobusińska, Michał Bartmański, Adam Zedler, Michał Studniarek

**Affiliations:** 1 Division of Oral Surgery, Medical University of Gdańsk, 7 Dębinki Street, 80-211 Gdańsk, Poland; adam.zedler@gumed.edu.pl; 2 Division of Orthodontics, Faculty of Medicine, Medical University of Gdańsk, 42c Aleja Zwycięstwa, 80-210 Gdańsk, Poland; marcin.stasiak@gumed.edu.pl; 3 University Dental Center, Medical University of Gdańsk, 1a Dębowa Street, 80-204 Gdańsk, Poland; natqus@gumed.edu.pl; 4 Institute of Manufacturing and Materials Technology, Faculty of Mechanical Engineering and Ship Technology, Gdańsk University of Technology, 11/12 Gabriela Narutowicza Street, 80-233 Gdańsk, Poland; michal.bartmanski@pg.edu.pl; 5 Department of Radiology, Faculty of Medicine, Medical University of Gdańsk, 17 Smoluchowskiego Street, 80-210 Gdańsk, Poland; michal.studniarek@gumed.edu.pl

**Keywords:** advanced platelet-rich fibrin, A-PRF, autografts, bisphosphonate-associated osteonecrosis of the jaw, bone disease, dentistry, growth factors, osteonecrosis of the jaw

## Abstract

Background: Medication-related osteonecrosis of the jaw (MRONJ) is drug-induced bone destruction that is exposed for a minimum of 6 to 8 weeks in patients who have not received head and neck radiotherapy and who have not been diagnosed with facial bone metastases. MRONJ treatment outcomes are unpredictable. Therefore, alternative treatment methods are being explored, such as blood-derived platelet-rich preparations enriched with growth factors, including advanced platelet-rich fibrin (A-PRF). The presence of growth factors may enhance healing and reduce post-procedure complications. There are no studies examining the effect of A-PRF on the healing of patients with MRONJ. The aim of this study was to retrospectively evaluate treatment outcomes of patients with MRONJ surgically treated without and with the use of A-PRF. Materials and methods: This retrospective study included 28 patients who suffered from osteomyelitis due to MRONJ and underwent surgical treatment between 2019 and 2024. The patients were divided into two groups: the first group received surgical treatment without A-PRF, and the second group received surgical treatment with the application of A-PRF. This study analyzed demographic and clinical data, as well as treatment outcomes. Results: The patients were aged from 43 to 82 years. The most common cause of MRONJ was the administration of zoledronic acid for oncological reasons (22 patients, 78.6%), given intravenously. In 20 patients (71.4%), the antiresorptive treatment lasted longer than three years. The obtained healing distribution was binomial (presence or absence of healing). Estimation of the probability of healing using the maximum likelihood method provided a result of approximately 64%. The probability of ten or more healed patients in the A-PRF group was 41%. A-PRF helps with a probability of 59%, and without A-PRF, it was lower. Concomitantly, the differences between the group with A-PRF and without A-PRF were not statistically significant. Conclusions: The patients with MRONJ should have regular check-ups with radiological examinations at least every six months to detect possible recurrence. Treatment for MRONJ is long and difficult. Treatment of non-advanced lesions, without additional risk factors (such as treatment with zoledronate intravenously for oncological purposes for 3 years), showed a better prognosis. Sometimes, in addition to surgery, it is necessary to consider alternative methods. A-PRF may enhance MRONJ healing. However, there is no evidence of a significant effect of A-PRF on the healing of MRONJ.

## 1. Introduction

**Figure 1:**
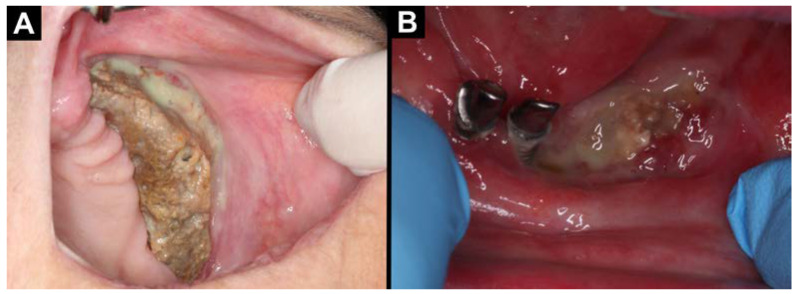
Intraoral photographs (**A**,**B**): (**A**) MRONJ located in the maxilla on the left side; (**B**) MRONJ located in the central part of the mandible.

Medication-related osteonecrosis of the jaw (MRONJ) refers to the destruction of jawbones caused by certain drugs. It occurs in patients undergoing or following treatment with antiresorptive or antiangiogenic drugs (e.g., bisphosphonates (BPs; alendronate, etidronate, ibandronate, clodronate, medronate, oxydronate, pamidronate, risedronate, tiludronate, and zoledronate), corticosteroids, denosumab (anti-RANKL), bevacizumab and aflibercept (anti-VEGFR), sunitinib, sorafenib, cabozatinib (tyrosine kinase inhibitors), sirolimus, everolimus (anti-mTOR), and adalimumab (anti-TNF)). In the early stage, avascular necrosis may occur and can only be detected by radiological examination. MRONJ involves the destruction of exposed bone (not covered by oral mucosa), with the exposure persisting for at least 6–8 weeks [1,2,3,4,5,6,7,8,9,10,11,12,13,14,15]. It develops in patients who have not undergone head and neck radiotherapy and do not have facial bone metastases (Figure 1) [1,2,3,10,11,12]. In patients receiving antiresorptive or antiangiogenic drugs, delayed wound healing in the oral cavity and osteonecrosis may occur after surgical procedures or due to other irritating factors, such as sharp edges of teeth or fillings, prosthetic restorations, tooth decay, periodontitis, bone fractures, exostoses, or a pronounced mylohyoid ridge. These complications are most often observed after teeth extractions. Drugs affecting bone turnover are used in the treatment of patients with bone metastases (most frequently for breast or prostate cancer), osteoporosis, fibrous dysplasia, osteogenesis imperfecta, otosclerosis, or multiple myeloma [2,4,7]. The risk of MRONJ is significantly higher in the cases undergoing treatment for tumor metastases when antiresorptive drugs are administered intravenously over 3 years and in those patients receiving additional therapies, such as chemotherapy, steroids, or thalidomide. Additional risk factors are comorbidities (e.g., diabetes, rheumatoid arthritis, calcium deficiency, and hyperparathyroidism) and tobacco smoking [7,10,11,12].

Oral irritants should be eliminated prior to initiating treatment with drugs that affect bone turnover. Dental caries must be treated, dentures adjusted, and damaged teeth removed to prevent MRONJ. Patients should undergo regular clinical and radiological examinations [10,11,12].

Osteonecrosis occurs approximately in 2/3 of cases in the mandible and in 1/3 in the maxilla. The main symptoms include exposed bone, often accompanied by purulent exudate, pain, soft tissue swelling, an active fistula (intra- or extraoral), halitosis, loosening or loss of teeth, numbness of the lip, a feeling of heaviness, and changes in tactile sensations. In advanced stages, pathological fractures of the mandible may occur. For an extended period, the affected area may remain asymptomatic [2,9,10,16,17,18,19,20,21].

The presence of osteonecrosis and other MRONJ signs can be assessed in radiological examinations, including dental X-rays, orthopantomography, cone beam computed tomography (CBCT), computed tomography (CT), scintigraphy, single-photon emission computed tomography (SPECT/CT), and positron emission tomography/computed tomography (PET/CT). Non-ionizing diagnostic methods, such as magnetic resonance imaging (MRI), are also utilized. Lesions may appear as radiolucent areas with osteolysis and osteosclerotic defects (commonly referred to as sequestrum with peripheral radiolucency). Unhealed tooth sockets are frequently observed. Pathological cracks, fractures, narrowing of the marrow space, cortical and trabecular bone destruction, and, sometimes, thickening and swelling of the periosteum and soft tissues can be identified on CBCT, CT, or MRI. On T1-weighted MRI, a loss of the fat signal in the bone marrow (typically present in the mandible) may be observed. In contrast, T2-weighted sequences show hypointensity in the bone marrow. Bone scintigraphy shows increased radionuclide uptake at the periphery of the lesion. Similarly, SPECT/CT reveals abnormal radionuclide uptake, such as technetium-99m methylene diphosphonate (99Tcm-MDP) or technetium-99m dicarboxypropane diphosphonate DPD (99Tcm-DPD). However, no uptake is observed in areas of necrosis. In PET/CT imaging, the uptake of fludeoxyglucose F18 (18F-FDG) is higher in inflamed areas compared to necrotic regions. In cases of aseptic necrosis, the affected area cannot be assessed due to the lack of inflammatory processes, which are essential for an accurate analysis [20,22,23,24,25,26].

Histopathological specimens reveal fragments of necrotic bone tissue containing bacteria. Necrotic bone is most commonly infected with *Actinomyces* sp., which are commensal bacteria in the oral cavity. The development of infections and actinomycosis is often associated with a decrease in the host’s immunity, commonly observed during oncological treatment. *Actinomycetes* sp. are Gram-positive (+) anaerobes. Bone necrosis creates an anaerobic environment because of vascular degradation and the formation of clots. *Actinomyces* sp. receive an ideal anaerobic environment for their existence. Other bacteria that have been successfully cultured from MRONJ include *Staphylococcus aureus*, *Escherichia coli*, *Streptococcus anginosus*, and *Pseudomonas mendocina* [19,27,28].

Treatment is mainly based on prevention, the elimination of risk factors, and, in advanced stages, on surgical treatment. MRONJ treatment outcomes are unpredictable [1,2,5]. Therefore, alternative treatment methods are being explored, such as blood-derived platelet-rich preparations enriched with growth factors, including advanced platelet-rich fibrin (A-PRF). A-PRF is a blood-derived platelet-rich preparation enriched with growth factors. It belongs to the third generation of preparations derived from blood. A-PRF was developed to obtain advanced blood derivatives rich in growth factors. Compared to classic PRF or L-PRF (leukocyte platelet-rich fibrin), its preparation involves a reduction in the centrifugation speed and a slight increase in the centrifugation time (A-PRF: 1500 rpm/14 min vs. PRF: 3000 rpm/12 min vs. L-PRF: 2700 rpm/12 min). As a result, the biological and physical properties of A-PRF are altered. It contains a greater number of leukocytes and platelets, and the resulting fibrin matrix is more cohesive and exhibits improved adhesion. Additionally, A-PRF is enriched with higher concentrations of cytokines, such as vascular endothelial growth factor (VEGF) and transforming growth factor beta (TGF-β), which enhance healing and stimulate tissue regeneration. An advancement of A-PRF is A-PRF+, which is produced at even lower speeds (1300 rpm/8 min), yielding a preparation with an even higher concentration of growth factors and neutrophils. The presence of growth factors may enhance healing and reduce post-procedure complications, which has been proven in many oral surgery procedures [29,30,31,32,33,34,35].

The aim of this study was to retrospectively evaluate the treatment outcomes of patients with medication-related osteonecrosis of the jaw, both without and with the use of advanced platelet-rich fibrin.

## 2. Materials and Methods

The retrospective study included 28 patients (43–82 years old) who suffered from osteomyelitis due to medication-related osteonecrosis of the jaw and underwent surgical treatment at the Division of Oral Surgery, University Dental Center of Medical University of Gdańsk, Poland, between 2019 and 2024. The retrospective research was carried out following the Declaration of Helsinki principles. Approval from the institutional ethics committee was obtained (Independent Bioethics Commission for Research, Medical University of Gdańsk, number of approval KB/510/2024). The patients signed an informed written consent to the surgical procedures.

The analysis considered several factors, including the patients’ age, gender, primary disease, duration of antiresorptive or antiangiogenic drug use (less than 3 years or 3 years and above), and route of drug administration (intravenous or oral). Additional factors included the presence of triggers for necrosis development (e.g., tooth extraction, periapical lesion), stage of the disease as classified by the American Association of Oral and Maxillofacial Surgeons (AAOMS, Table 1), and the extent of the lesions in the clinical and radiological imaging (less than 3 cm and 3 cm and above). The analysis also evaluated the type of treatment applied: either surgical removal of the necrotic bone alone or combined with the local administration of growth factors derived from the patient’s blood. Treatment success and the duration of the follow-up were assessed. Additionally, several factors influencing a worse prognosis were evaluated, including the use of zoledronic acid, intravenous administration, treatment duration of at least three years, a previous episode of jawbone necrosis, and oncological treatment.

Two groups of patients were compared. The first group comprised patients who received surgical treatment involving the removal of necrotic lesions. The second group included patients who, in addition to the removal of necrotic lesions, received advanced platelet-rich fibrin.

### 2.1. Inclusion Criteria

The study included adult patients with MRONJ who had been treated with antiresorptive or antiangiogenic drugs. The eligibility criteria required bone exposure persisting for a minimum of 6–8 weeks, the absence of prior head and neck radiotherapy, and no diagnosis of facial bone metastases. Surgical procedures were performed only on patients in good general condition with normal results in basic diagnostic tests (e.g., blood morphology).

### 2.2. Exclusion Criteria

The study excluded patients with osteonecrosis of the jaw who had not been treated with antiresorptive or antiangiogenic drugs, whose bone exposure persisted for less than 6–8 weeks, who had undergone head and neck radiotherapy, or who were diagnosed with facial bone metastases. Additionally, underage patients and those who did not provide consent for treatment were excluded.

### 2.3. Surgical Procedure

Before the treatment, extraoral and intraoral examinations and radiological diagnostic imaging were performed. Orthopantomography or cone beam computer tomography was assessed using the CS 3D Imaging v3.5.18 software (Carestream Health Inc., Trophy, Croissy-Beaubourg, France). Each patient received 1 g of amoxicillin with cluvuate acid (0.875 g + 0.125 g; Augmentin, GlaxoSmithKline, London, UK) every 12 h one day before the procedure. In patients with allergies, 300 mg of clindamycin was administered every 8 h starting the day before the surgery (Dalacin C, Pfizer, Brooklyn, NY, USA). The antibiotic therapy protocol was implemented according to the Recommendations of the Working Group of the Polish Dental Association and the National Antibiotic Protection Program regarding the use of antibiotics in dentistry [36]. Additionally, the patients rinsed their mouths with a 0.1% chlorhexidine solution (Eludril Classic, Pierre Fabre Oral Care, Lavaur, France).

In the group of patients who received A-PRF before the surgery, 4 tubes of 10 mL venous blood were collected into sterile, anticoagulant-free, glass-coated plastic tubes. After that, the tubes were immediately centrifuged (All Centrifuge, Scilogex, LLC, Rocky Hill, CT, USA). The blood was centrifuged at 1500 rpm for 14 min. Then, the A-PRF clots were put into a PRF box (Quadrostom, Kraków, Poland), and A-PRF plugs were made.

**Figure 2:**
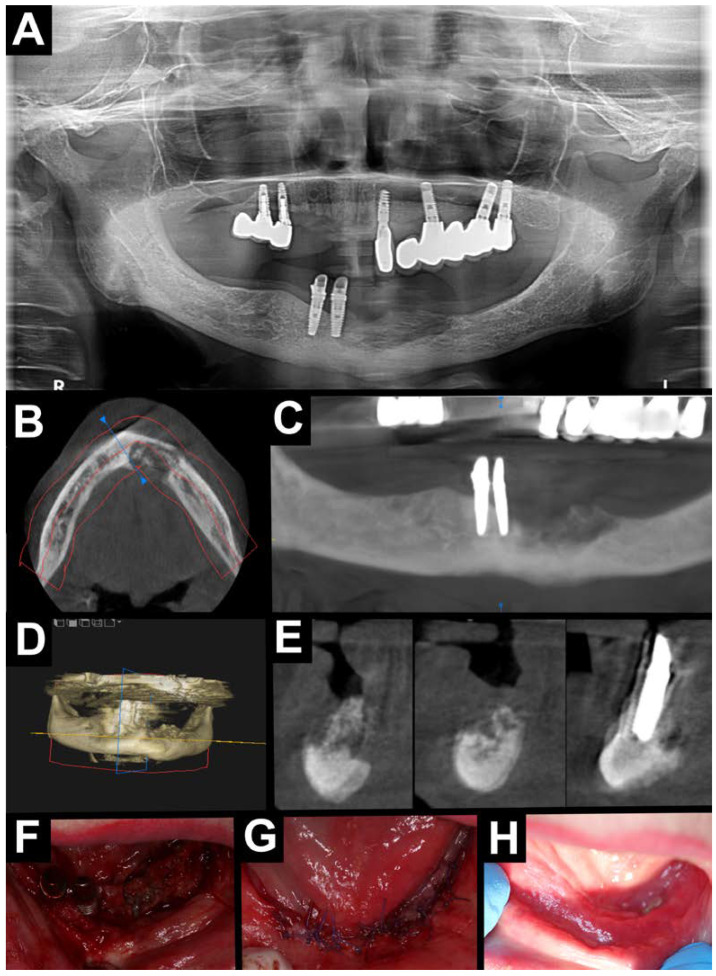
Treatment without A-PRF. Radiological diagnostic imaging of MRONJ (**A**–**E**). (**A**) Orthopantomography—area with osteolysis and osteosclerosis located in the mandible on the anterior area; CBCT (**B**–**E**). (**B**) Axial view—area with osteolysis and osteosclerosis on the anterior area of the mandible. (**C**) Orthopantomographic reconstruction—area with osteolysis and osteosclerosis on the anterior area of the mandible. (**D**) Pseudo-3D reconstruction—area with osteolysis and osteosclerosis on the anterior area of the mandible. (**E**) Cross-sectional view—area with osteolysis and osteosclerosis on the anterior area of the mandible. Intraoral photographs (**F**–**H**). (**F**) Visible necrotic bone after mucoperiosteal flap elevation. (**G**) After suturing the wound without tension. (**H**) Healing after 2 weeks (red line—panoramic reconstruction site; yellow line – cross-sectional area line; blue line—cross-sectional area line).

**Figure 3:**
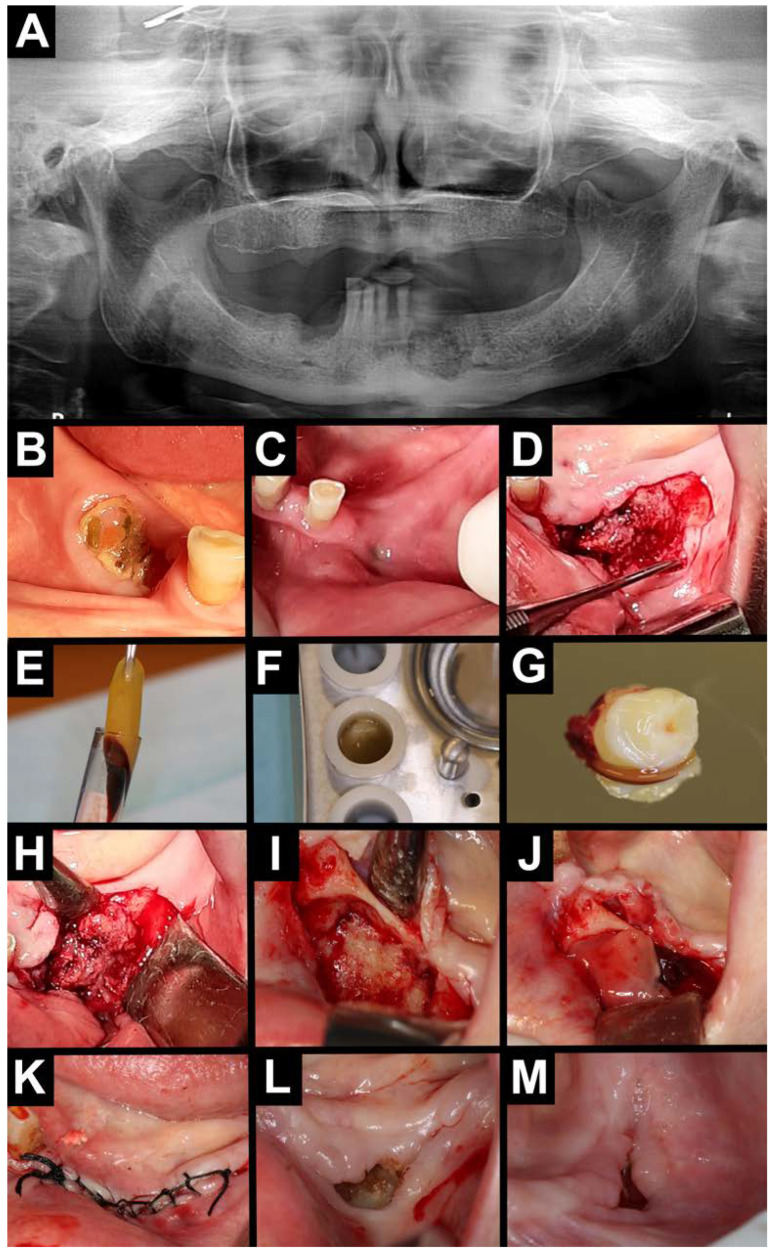
Treatment with A-PRF. (**A**) Orthopantomography—area with osteolysis and osteosclerosis on the left and right sides of the mandible. Intraoral photography (**B**–**D**). (**B**) Exposed necrotic bone on the right side of the mandible. (**C**) Intraoral purulent fistula on the left side of the mandible. (**D**) Visible necrotic bone after mucoperiosteal flap elevation on the left side of the mandible. A-PRF preparation (**E**–**G**). (**E**) A-PRF clot. (**F**) A-PRF in a PRF box. (**G**) A-PRF plug. Intraoral photographs (**H**–**M**). (**H**,**I**) Necrotic tissue was removed, and the bone was bled. (**J**) A-PRF plug placed intra-wound. (**K**) Wound was sutured. (**L**) Healing after 2 weeks. (**M**) Healing after 6 weeks.

The surgical procedure was performed under local anesthesia administered via injection (Figure 2 and Figure 3). The type of anesthesia depended on the location of the lesion. The nerves were blocked using 4% articaine hydrochloride with 1:100,000 epinephrine (Septodont, Lancaster, PA, USA). A 15c scalpel blade (Swann-Morton, Sheffield, UK) was used, and the mucoperiosteal flap was raised. The epithelialized edges of the mucosa were prepared and debrided. The necrotic bone was curetted, fixed in 10% formalin, and submitted for histopathological examination. The bottom of the lesion was cleaned down to the bleeding bone using a rose head bur (Meisinger, Hager, and Meisinger GmbH, Neuss, Germany) mounted on a surgical straight handpiece (S-11 L G, W&H Dentalwerk Bürmoos GmbH, Bürmoos, Austria) at 40,000 rpm, with abundant irrigation using 0.9% NaCl. The wound was rinsed with 20 mL of 0.5% metronidazole solution (Polpharma SA, Starogard Gdański, Poland). In the A-PRF group, four wound plugs were placed at this stage. The wound was sutured without tension, and hemostasis was achieved. A gauze pad was applied, and the patient was instructed to bite on it and hold it in place for 20 min.

Patients continued antibiotic therapy for 14 days in the high-risk group (defined as receiving zoledronic acid, intravenous bisphosphonates, or the drug for at least 3 years, or those with a previous episode of jawbone inflammation or necrosis) and for 7 days in the low-risk group (not meeting these criteria) [36]. The sutures were removed after 14 days.

### 2.4. Post-Operative Evaluation and Follow-Up

Patients attended regular follow-up appointments: initially at 2 and 6 weeks, then every 3 months, and annually if the lesions healed properly. For non-healing lesions, the frequency of visits was adjusted on an individual basis.

### 2.5. Statistical Analysis

A statistical analysis was performed using the R software (version 4.4.2, the R Foundation for Statistical Computing Platform, Boston, MA, USA). Patients’ age, gender, primary disease, duration of antiresorptive or antiangiogenic drug use (less than 3 years or 3 years and above), route of drug administration (intravenous or oral), the presence of triggers for necrosis development (e.g., tooth extraction, periapical lesion), stage of the disease as classified by the AAOMS, the extent of the lesions in the clinical and radiological imaging (less than 3 cm and 3 cm and above), the type of treatment applied, treatment success, and the duration of follow-ups were quantitatively summarized. Statistical analyses were performed using the maximum likelihood method. The non-parametric ANOVA test was used for a comparative analysis. Statistically significant results were accepted for *p* ≤ 0.05.

## 3. Results

### 3.1. Patient Characteristics

A total of 28 patients with MRONJ were included in this study. At the time of the MRONJ diagnosis, the patients were aged between 43 and 82 years, with a mean age of 70.2. The female group was larger than the male group (*n* = 21, 75% vs. *n* = 7, 25%).

Eleven patients (39.3%) had multiple myeloma, seven (25%) had breast cancer, four (14.3%) had prostate cancer, four (14.3%) had osteoporosis, and two (7.1%) had other. All patients were treated exclusively with bisphosphonates—either zoledronic acid or alendronic acid. The most common cause of MRONJ was the administration of zoledronic acid for oncological indications (*n* = 22, 78.6%). Zoledronic acid was administered intravenously (100% of cases), and alendronic acid was given only orally. In 20 patients (71.4%), the antiresorptive treatment lasted longer than 3 years.

The factors contributing to MRONJ included surgical procedures or infection in the oral cavity. These were tooth extraction (*n* = 25, 89.3%), inflammatory lesions such as periapical lesions or periodontal disease (*n* = 2, 7.1%), or the presence of dental implants (1/3.6%).

The location in the mandible was more common than in the maxilla (*n* = 24, 82.1% vs. *n* = 5, 17.9%). All patients were diagnosed with stage 3 according to the AAOMS classification. All patients had an intraoral fistula, and two also had an extraoral fistula. In most cases, the lesion size was up to 3 cm (*n* = 22, 78.6%). Among the oncological patients, lesions up to 3 cm occurred in 100% of the patients in the A-PRF group, and in the group without A-PRF, they were 73%.

Among the non-oncological patients, lesions up to and above 3 cm occurred in the same number of patients in both groups (three patients each). In the A-PRF group, healing was complete in 100% of the patients, and in the non-A-PRF group, it was 66.8%. The average follow-up period was 14.79 months. The demographic and clinical characteristics, divided into groups with and without A-PRF treatment, are presented in Table 2.

### 3.2. Comparison of the Impact of A-PRF Use in Relation to Demographic and Clinical Features

The use of A-PRF was compared in relation to demographic and clinical features. No statistical significance was found for the healing and location of lesions (mandible vs. maxilla, *p* = 0.4373) or the size of the lesion (less than 3 cm vs. 3 cm and above, *p* = 0.8957). Statistical significance was obtained only for the presence of healing and the number of additional risk factors (1–5), including zoledronic acid use, intravenous drug administration, drug use for at least three years, a previous episode of jawbone necrosis, or oncological treatment (*p* = 0.006989).

The obtained healing distribution was binomial (presence or absence of healing). Estimation of the probability of healing using the maximum likelihood method provided a result of approximately 64%. The probability of ten or more healed patients in the A-PRF group was 41%. A-PRF helps with a probability of 59%. Concomitantly, the differences between the group with A-PRF and without A-PRF were not statistically significant (*p* = 0.449).

## 4. Discussion

For patients planned to receive oncological or osteoporosis treatment with drugs associated with MRONJ, prophylactic management should be pivotal. Therapy with antiresorptive and antiangiogenic drugs should be started only after eliminating the infection foci in the oral cavity. The patient should undergo a clinical and radiological evaluation or at least an orthopantomographic examination. In complex cases, such as previous endodontic treatments, periodontal disease assessments, or completely impacted teeth, additional tissue evaluation in CBCT may be necessary [10,11,12].

Due to the challenges in treating medication-related osteonecrosis of the jaw, various therapy approaches are undertaken, including non-surgical and surgical treatments, as well as a combination of both. Alternative techniques are also explored. According to the American Association of Oral and Maxillofacial Surgeons, in the initial stage (stage 0) of the disease, the primary goal is to eliminate irritating factors. Patients are advised to rinse their mouths with 0.12% chlorhexidine. Antibiotic treatments, typically amoxicillin with clavulanic acid or clindamycin, are recommended in the cases of infection. Pain and inflammation management includes analgesics and anti-inflammatory medications. For patients with mucosal damage but no symptoms of infection (stage I), treatment follows the same protocol as in stage 0. In the case of pain, low-level laser therapy (LLLT) can be introduced for biomodulation. In stage II, where bone exposure is accompanied by an active infection, antibacterial, anti-inflammatory, and antiseptic rinses are necessary. At this stage, radiological examination often reveals bone lysis with sequestration. Surgical treatment should be considered as a last resort, as it may exacerbate the lesion progression. Alternatively, a superficial MRONJ debridement can be performed. In the advanced stage (III), surgical treatment is necessary. In stage III, intraoral and extraoral purulent fistulas and, in some cases, pathological fractures are observed. Surgical procedures include sequestrum removal, bone debridement down to healthy bone, or segmental resection [1,2,4,34,35]. In addition to LLLT, other supportive therapies include hyperbaric oxygen therapy (HOT), photobiomodulation, ozone therapy, blood-derived platelet-rich preparations enriched with growth factors, and recombinant human bone morphogenetic proteins (rhBMP). Additional pharmacological treatments involve teriparatide, pentoxifylline, and vitamins E and D. However, alternative methods should not be used as monotherapy but rather as part of a multimodal treatment approach [34,35,37,38,39,40,41,42,43,44].

A-PRF is an autogenous, non-allergenic, and non-immunogenic blood-derived platelet-rich preparation enriched with growth factors. Due to the presence of growth factors, it promotes healing and helps reduce postoperative complications. Wound healing is supported by the gradual release of growth factors (for up to 4 weeks), which enhances regenerative processes, such as cell proliferation and differentiation—particularly of fibroblasts, endothelial cells, and osteoblasts—crucial for both soft and hard tissue regeneration. It also stimulates neovascularization, modulates inflammation, and accelerates the formation of epithelium and connective tissues [29,30,31,32,33,34,35].

The available studies about platelet-rich preparations are case reports [45,46,47,48,49] or were used in prophylaxis (e.g., filling the post-extraction socket) [50,51,52,53,54,55] or other blood products—PRF [46,51,53,56,57,58] and L-PRF [45,47,48,52,54,59,60,61,62,63,64]. A-PRF has been used in an animal model [65] and in one case report article [66]. Three original publications analyzed A-PRF+, not A-PRF, as it differs in the production process compared to A-PRF [49,67,68]. In the available studies, the authors reported that the addition of blood-derived agents was a valuable addition, supported healing, and even led to the complete healing of lesions. In a study using PRF, de Almeida Barros Mourão et al. [56] achieved complete treatment success, while in the case of L-PRF, Zelinka et al. [59]—85–94%, Özalp et al. [60]—69%, Aslam et al. [62]—100%, and Yalcin-Ülker [63] achieved a success rate of 80%.

To date, our study is the only one to have investigated A-PRF and its effect on healing in patients with MRONJ. More than 85% of the study participants were oncology patients treated with zoledronic acid administered intravenously. Moreover, the lesions were highly advanced, classified as stage III according to AAOMS. This could have influenced the treatment outcomes. In our study, whether the patient was an oncology patient or not had no impact on the healing process at such advanced stages, regardless of the use of A-PRF. Referring to studies analyzing A-PRF+ (not A-PRF), Giudice et al. [49] reported that 74.5% of their study group consisted of oncology patients, with a predominance of stage II cases according to AAOMS (53%) over stage III (47%). Their findings indicated that A-PRF+ improved the patients’ quality of life in the first month after the procedure, reducing both pain and the risk of post-procedure infections. This relationship was not observed in later follow-up periods. Blatt et al. [68] also primarily evaluated oncology patients (84.7%); however, the majority (78.8%) had AAOMS stage I disease, while only 1.9% had stage III disease. Their study found no statistical correlation between healing and the use of A-PRF+. Roman et al. [67] had a heterogeneous study group, including patients with MRONJ who had undergone radiotherapy and cases with a combination of MRONJ and radiotherapy. Among patients with MRONJ, 60.5% were taking oral antiresorptive drugs, which is considered a lower-risk group for MRONJ based on current knowledge. Regarding MRONJ staging, 42.5% of patients were classified as AAOMS stage II, 18.5% as stage I, and 3.7% as stage III. This study concluded that A-PRF+ may serve as an adjunctive method to support wound healing; however, no correlation was found with reductions in disease severity, pain, or oral health-related quality of life.

The early and accurate diagnosis of medication-related osteonecrosis of the jaw is pivotal. Our article broadens the knowledge to include the latest advances in diagnosis and treatment of MRONJ. This article presents diagnostic methods as well as a simple and cheap technique of treating MRONJ using A-PRF. Most importantly, it is non-allergenic and does not cause immunological reactions. Biocompatible healing and the pursuit of nature is called biomimetic treatment. Many patients may require this type of treatment from their doctor due to their beliefs or religion.

The study was limited by the relatively small study group, and the treatment was used only in the advanced stages (which could have resulted in worse treatment results). It would be valuable to compare two groups that are homogeneous in terms of the number of participants and the stage of disease progression but differ in the indication for antiresorptive therapy—oncologic versus non-oncologic patients. A significantly better response to A-PRF treatment was observed in the case of small lesions. Some patients experienced progression of the neoplastic disease, which resulted in a deterioration of their general condition and could have contributed to possible healing failure in the group with or without A-PRF.

Future research directions should focus on large cohort studies. It is important to remember to support the treatment with antibiotic therapy. An interesting solution would be a combined treatment with pentoxifylline and tocopherol, then performing a surgical procedure—bone debridement with additional bone ablation with a laser and the application of growth factors in an antibiotic cover, followed by laser biostimulation.

## 5. Conclusions

The patients with medication-related osteonecrosis of the jaw should undergo regular check-ups, including radiological examinations, at least every six months to detect possible recurrence. MRONJ treatment is prolonged and challenging. Non-advanced lesions, in the absence of additional risk factors (such as zoledronate treatment administered intravenously for oncological reasons over a period of more than 3 years), tend to have a better prognosis. In some cases, alternative therapies may support surgical treatments. A-PRF may enhance MRONJ healing. However, there is no evidence of a significant effect of A-PRF on the healing of MRONJ. We believe that increasing awareness and understanding of these therapeutic approaches is essential. Further studies on the use of A-PRF in the treatment of MRONJ are needed in a homogeneous, large study group in oncological and non-oncological patients.

## Figures and Tables

**Table 1:** Classification of MRONJ by the American Association of Oral and Maxillofacial Surgeons.

Stage 0	- Nonexposed bone - Non-specific clinical symptoms (odontalgia, dull pain in maxilla-facial bones/sinuses, altered neurosensory function, teeth loosening, intra- or extraoral swelling) - Changes in radiography (bone resorption not compared with periodontal disease, osteosclerosis)
Stage 1	- Exposed bone or the presence of a purulent fistula - Presence of clinical symptoms - Radiographic changes (bone resorption not attributable to periodontal disease or areas of osteosclerosis)
Stage 2	- Exposed bone or purulent fistula - Presence of clinical symptoms - Changes in radiography (bone resorption not compared with periodontal disease, osteosclerosis)
Stage 3	- Exposed bone or a purulent fistula extending to the alveolar bone - Pathological fractures - Oroantral or oronasal communications or fistulas - Osteolysis extending to the inferior border of the mandible

**Table 2:** Demographic and clinical features divided into two groups: surgically treated and surgically treated with A-PRF application.

Features		Surgical Treatment	Surgical Treatment and Application of A-PRF	*p* Value
Gender	Female	12 (42.9%)	9 (32.1%)	0.2043
	Male	2 (7.1%)	5 (17.9%)	
Average age at MRONJ diagnosis	Female	72.3 years	79.6 years	-
	Male	71 years	64.1 years	
Mean age at MRONJ diagnosis		72.07 years	68.29 years	0.3928
Primary disease	Oncological disease	11 (39.3%)	11 (39.3%)	0.6369
	Other diseases	3 (10.7%)	3 (10.7%)	
Type of disease	Multiple myeloma	3 (10.7%)	8 (28.6%)	0.0383
	Breast cancer	5 (17.9%)	2 (7.1%)	
	Prostate cancer	3 (10.7%)	1 (3.6%)	
	Osteoporosis	2 (7.1%)	2 (7.1%)	
	Other	1 (3.6%)	1 (3.6%)	
Type of drug	Zoledronic acid	11 (39.3%)	11 (39.3%)	0.1708
	Alendronic acid	3 (10.7%)	3 (10.7%)	
	Other	0 (0%)	0 (0%)	
Duration of antiresorptive or antiangiogenic drug use	Less than 3 years	6 (21.4%)	2 (7.1%)	0.3097
	3 years and above	8 (28.6%)	12 (42.9%)	
Route of administration	Intravenous	11 (39.3%)	11 (39.3%)	0.7993
	Oral	3 (10.7%)	3 (10.7%)	
Presence of triggers for necrosis development	Yes	14 (100%)	14 (100%)	0.4490
	No	0 (0%)	0 (0%)	
Localization of MRONJ	Mandible	12 (42.9%)	11 (39.3%)	0.6369
	Maxilla	2 (7.1%)	3 (10.7%)	
Size of lesion	Less than 3 cm	10 (35.7%)	12 (42.9%)	0.3757
	3 cm and above	4 (14.3%)	2 (7.1%)	
Treatment success	Yes	8 (28.6%)	10 (35.7%)	0.4489
	No	6 (21.4%)	4 (14.3%)	
Mean follow-up period		11.1 months	18.1 months	0.0521

## Data Availability

The original contributions presented in the study are included in the article, further inquiries can be directed to the corresponding author.

## Abbreviations

99Tcm-DPD	technetium-99m dicarboxypropane diphosphonate
99Tcm-MDP	technetium-99m methylene diphosphonate
A-PRF	advanced platelet-rich fibrin
A-PRF+	advanced platelet-rich fibrin +
AAOMS	American Association of Oral and Maxillofacial Surgeons
anti-mTOR	anti-mammalian target of rapamycin
anti-RANKL	anti-receptor activator of nuclear factor-kappaB ligand antibody
anti-VEGFR	anti-vascular-endothelial growth factor receptor
BPs	bisphosphonates
CBCT	cone beam computed tomography
F18-FDG	fludeoxyglucose F18
HOT	hyperbaric oxygen therapy
L-PRF	leukocyte platelet-rich fibrin
LLLT	low-level laser therapy
MRI	magnetic resonance imaging
MRONJ	medication-related osteonecrosis of the jaw
PET/CT	positron emission tomography/computed tomography
PRF	platelet-rich fibrin
rhBMP	recombinant human bone morphogenetic proteins
SPECT/CT	single-photon emission computed tomography
